# Addition of rituximab in relapsed/refractory chronic lymphocytic leukemia after progression on venetoclax monotherapy

**DOI:** 10.1002/jha2.177

**Published:** 2021-03-03

**Authors:** Sasanka Handunnetti, Mary Ann Anderson, Andrew W. Roberts, Matthew S. Davids, Shuo Ma, Michelle Boyer, Jennifer Arzt, Abdullah Al Masud, Relja Popovic, Amanda Jacobson, Su Y. Kim, John F. Seymour

**Affiliations:** ^1^ Clinical Haematology Department Peter MacCallum Cancer Centre and Royal Melbourne Hospital Melbourne Victoria Australia; ^2^ Faculty of Medicine, Dentistry and Health Sciences University of Melbourne Melbourne Victoria Australia; ^3^ Division of Blood Cells and Blood Cancer Walter and Eliza Hall Institute of Medical Research Parkville Victoria Australia; ^4^ Department of Medical Oncology Dana‐Farber Cancer Institute Boston Massachusetts USA; ^5^ Robert H. Lurie Comprehensive Cancer Center Northwestern University Chicago Illinois USA; ^6^ F. Hoffmann‐La Roche Ltd Welwyn Garden City UK; ^7^ AbbVie, Inc. North Chicago Illinois USA

**Keywords:** chronic lymphocytic leukemia, relapsed/refractory, rituximab, small lymphocytic lymphoma, venetoclax

## Abstract

Venetoclax is approved as monotherapy and in combination with rituximab for relapsed/refractory (R/R) chronic lymphocytic leukemia (CLL). Two Phase 1 studies (M12‐175 [NCT01328626]; M13‐365 [NCT01682616]) were conducted in which patients who initially responded and then progressed on venetoclax monotherapy could receive added rituximab. Ten patients were evaluated (M12‐175, n = 8; M13‐365, n = 2), and five (50%) responded again upon addition of rituximab, including three complete and two partial responses. Responses were ongoing after 5–10 months of follow‐up. Addition of rituximab was well tolerated. These findings indicate potential clinical benefit with rituximab added to venetoclax post‐progression in some patients with R/R CLL.

## 1 INTRODUCTION

Venetoclax is a selective B‐cell lymphoma‐2 inhibitor approved for the treatment of patients with chronic lymphocytic leukemia (CLL)/small lymphocytic lymphoma (SLL) in the frontline and relapsed settings as monotherapy or in combination with an anti‐CD20 antibody [1]. Although responses are achieved in approximately 75% of patients with monotherapy in the relapsed/refractory (R/R) setting, most patients will develop progressive disease (PD) while on continuous venetoclax after a median duration of 3 years [2, 3]. Including rituximab from the outset induces high response rates (92%) and more frequent deep responses when indirectly compared with venetoclax monotherapy in patients with R/R CLL/SLL [4]; however, it is unknown whether the later addition of rituximab after PD on venetoclax can improve outcomes.

The primary analyses of two phase 1 dose‐escalation studies of venetoclax in R/R CLL/SLL (M12‐175 and M13‐365) have been previously reported [5, 6]. During long‐term follow‐up in these studies, patients who progressed on venetoclax monotherapy could, at investigator discretion, receive added rituximab while continuing venetoclax [5, 6]. Here, we report efficacy and safety outcomes for these patients.

## 2 METHODS

M12‐175 (NCT01328626; data cutoff July 15, 2019) and M13‐365 (NCT01682616; data cutoff June 4, 2019) were phase 1 open‐label dose‐escalation studies conducted in accordance with the Declaration of Helsinki and all applicable local guidelines. Both trials were approved by the ethics committees at each participating institution. All patients provided written informed consent. Patients aged ≥18 years with R/R CLL/SLL and an Eastern Cooperative Oncology Group performance status score ≤1 were enrolled. Full inclusion criteria have been previously described [5, 6].

In M12‐175, 56 patients received venetoclax with a stepwise dose ramp‐up to reach target doses of 200–1200 mg once daily in dose escalation, and 60 patients were enrolled in an expansion cohort at the recommended phase 2 dose (RP2D) of 400 mg once daily continuously until progression [5]. In M13‐365, 49 patients received venetoclax daily, using a stepwise weekly escalation schedule, to target doses of 200–600 mg once daily continuously and rituximab for 6–9 doses, with most patients following a schedule of 375 mg/m^2^ in month 1 and 500 mg/m^2^ in months 2–6 [6]. In both studies, responses were evaluated based on the International Workshop on CLL (iwCLL 2008) criteria [7]. Minimal residual disease (MRD) testing was performed in bone marrow (BM) and peripheral blood (PB) using ≥4‐color flow cytometry, with a minimum sensitivity of 0.01% per European Research Initiative on CLL methodology [8].

Per protocol amendments, patients with CLL PD while on venetoclax monotherapy could remain on venetoclax and receive rituximab (375 mg/m^2^ followed by 500 mg/m^2^ monthly for 5 months) after PD, per iwCLL criteria [7]. Tumor lysis syndrome (TLS) prophylaxis was not mandated for rituximab treatment after progression; low‐risk standard TLS prophylaxis was followed for some patients. Adverse events (AEs) were graded per the National Cancer Institute Common Terminology Criteria for Adverse Events. In this patient series, we recorded clinicopathological factors both at study commencement and at the time of CLL progression. We evaluated responses and described the duration of clinical benefit following rituximab treatment. AEs occurring after starting additional rituximab were summarized.

## 3 RESULTS AND DISCUSSION

Adding rituximab to continuous venetoclax therapy after documented CLL progression was not mandatory per study protocols and occurred at investigator discretion. The case studies (**Supplementary Materials**) include patients who had achieved durable responses to venetoclax‐based therapy, had indolent CLL relapse on venetoclax monotherapy, and opted to continue therapy and receive rituximab. Ten patients (M12‐175: n = 8; M13‐365: n = 2) had rituximab added to ongoing venetoclax after developing PD per iwCLL criteria (*n* = 9) or rising MRD in PB (>1 log increase; *n* = 1) while on continuous venetoclax monotherapy. Progression was characterized by lymphadenopathy in seven of 10 patients, although none had bulky disease; the median of the largest node size was 3.2 cm (range, 2.0–3.9 cm). No patient had clinically aggressive relapse or Richter transformation. In one of six evaluable patients in this series, an emergent *BCL2* mutation (Gly101Val) was detected at CLL progression; the mutation in this patient was the major clone and has been previously described [9].

**FIGURE 1:**
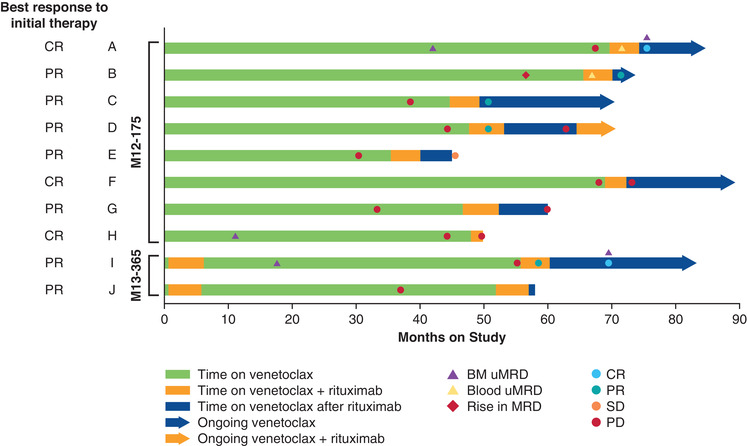
Best response, MRD response, and time on study drug Abbreviations: BM, bone marrow; CR, complete response; MRD, minimal residual disease; PD, progressive disease; PR, partial response; SD, stable disease; uMRD, undetectable minimal residual disease.

The characteristics of this cohort and responses after adding rituximab to venetoclax therapy are summarized in Table 1 and represented in a swimmer plot (Figure 1). Of the M12‐175 subset, the best initial response to venetoclax monotherapy was complete response (CR) in three of eight patients and partial response (PR) in five of eight patients. The median time from drug commencement to progression on venetoclax monotherapy was 44 months (range, 30–68). Clinical benefit was observed upon adding rituximab to venetoclax monotherapy, with four of eight patients achieving new responses (one CR, three PRs). One of the four responders again developed CLL progression 7 months after rituximab response and subsequently achieved a third response to a second course of additional rituximab treatment. Another responder with a *BCL2* mutation detected at CLL PD conferring putative venetoclax resistance [9], achieved MRD‐negative CR on completion of rituximab therapy. This mutation (Gly101Val) was found to reduce sensitivity to venetoclax in vitro; however, full resistance requires additional changes to the microenvironment, which varies between patients [9]. As of the data cutoff, CLL response was ongoing in all four patients, providing additional clinical benefit ranging from 5.4+ to 9.8+ months; one patient developed PD after database cutoff. Four patients did not achieve a response following rituximab treatment; one patient who has not yet had a formal response assessment remains on venetoclax monotherapy with stable disease with ongoing clinical benefit as judged by the treating hematologist.

**TABLE 1:** Best response on venetoclax monotherapy and after combination with rituximab

	Patient	Age^a^ (year)	Prior therapies (number)	17p Del and/or *TP53* Mut	Venetoclax dose (mg)^b^	Best response to initial therapy (MRD)	Time to progression on initial therapy (mo)/Type of progression	Best response to venetoclax + rituximab (MRD)	Second response duration (mo)^c^	Total time on venetoclax therapy (mo)^d^
**M12‐175**	**A**	70	3	Yes	600	CR (PB uMRD)	67.1/marrow	CR (BM uMRD)	5.9 Ongoing	82.4
	**B**	66	2	No	400	PR (PB MRD+)	56.2/marrow^e^	PR (PB uMRD)	5.6 Ongoing	71.6
	**C**	69	2	N/A	400	PR (N/A)	38.2/nodal	PR (N/A)	5.4 Ongoing	68.1
	**D**	61	1	N/A	400	PR (BM/PB MRD+)	44.0/nodal	PR (BM MRD+/ PB uMRD)	7.0 (2.8 Ongoing)^f^	68.2
	**E**	71	3	Yes	400	PR (BM/PB MRD+)	30.2/nodal	SD (N/A)	N/A	44.6
	**F**	62	4	No	300	CR (BM/PB MRD+)	67.7/marrow	PD (N/A)	N/A	87.2
	**G**	58	2	No	400	PR (N/A)	32.9/nodal	SD (N/A)	N/A	59.7
	**H**	64	6	No	400	CR (BM uMRD)	44.0/nodal	PD (N/A)	N/A	49.6
**M13‐365**	**I**	50	5	No	200/400	PR (uMRD)	54.0/nodal	CR (BM uMRD)	23 Ongoing	80.4
	**J**	62	3	Yes	400	PR (BM MRD 1.68%)	37.0/nodal and PB lymphocytosis	PD	N/A	58.3

Abbreviations: BM, bone marrow; CR, complete response; del, deletion; F, female; M, male; MRD, minimal residual disease; mut, mutated; N/A, not available; PB, peripheral blood; PD, progressive disease; PR, partial response; SD, stable disease; uMRD, undetectable minimal residual disease.

Data cutoff dates were July 15, 2019 for M12‐175 and June 4, 2019 for M13‐365.

Patients A, B, D, E, and F did not have complex karyotype (presence of ≥3 chromosomal aberrations [structural and/or “numeric”] identified by chromosome banding analysis); data were unavailable for patients C and G through J.

All patients had received anti‐CD20 monoclonal antibody as prior therapy.

^a^ Age at study entry.

^b^ Predominant dose of venetoclax received is listed. The following patients also received other doses of venetoclax on study: patient A had reduction to 600 mg from 800 mg due to trial directive; patients B and D had venetoclax dose increased to 600 mg due to worsening response. Patients I and J were initially enrolled in dose‐escalation cohorts < 400 mg once daily and received 400 mg venetoclax when it was established as the recommended phase 2 dose and increased to 600 mg venetoclax after progression. Patient I received 200 mg and 400 mg venetoclax for equal amounts of time.

^c^ Defined as the time from PR or better after rituximab treatment to data cutoff or progression.

^d^ Last dose of venetoclax or data cutoff dates were used.

^e^ Patient did not experience PD per International Workshop on Chronic Lymphocytic Leukemia criteria but had MRD increase.

^f^ Reports the response duration after the first round of rituximab (time from BM/PB MRD+ PR to progression) and the second round of rituximab (PB uMRD) to data cutoff.

Both patients in M13‐365 who initially received venetoclax in combination with rituximab achieved PR to initial therapy in dose escalation (dose < 400 mg once daily) and subsequently received 400 mg once daily after the RP2D was determined. These patients developed PD at 36 and 55 months, and both received an increase in venetoclax dose to 600 mg without CLL response. One patient achieved a PR shortly after being re‐treated with rituximab, then attained CR with undetectable MRD in BM 12 months later, with ongoing undetectable MRD. The second patient did not achieve disease response following rituximab re‐treatment and discontinued the study due to PD.

No significant safety findings were observed with the addition of rituximab to ongoing venetoclax monotherapy. Treatment‐emergent AEs related to rituximab occurred in three of 10 patients, including grade 2 infusion reaction, grade 1 fatigue, and one patient with grade 2 neutropenia and grade 2 upper respiratory tract infection. No biochemical or clinical TLS with addition of rituximab was reported. Other AEs of interest post‐rituximab included grade 3/4 neutropenia in two patients, one event of grade 3 *Campylobacter* infection, and one event of grade 3 pneumonia.

Despite the durable responses that can be achieved by venetoclax with rituximab in the R/R setting [10], options for treating patients with CLL who progress on venetoclax are limited. Bruton tyrosine kinase inhibitors (BTKis) can be effective in BTKi‐naive patients [11, 12] or in patients ceasing initial BTKi therapy because of intolerance; however, interventions that prolong the clinical benefit of venetoclax are desirable. One option for achieving this is to re‐treat patients with rituximab or other anti‐CD20 antibodies such as obinutuzumab, which has been shown to be a more effective antibody to partner with chemotherapy than rituximab in the frontline setting [13, 14] and has an acceptable safety profile with venetoclax [15]. In this era of novel CLL therapies [4, 16], this case series provides insight into the potential benefit of rituximab in salvaging the loss of response to venetoclax. The addition of rituximab could be an effective and tolerable therapeutic strategy for some patients with CLL who develop PD after previous durable responses to venetoclax monotherapy. These findings suggest that continuing venetoclax and re‐treating with rituximab may extend the clinical utility of venetoclax in some patients. Although the cost of such an approach may be limiting for some, this option could be particularly useful for patients with otherwise limited treatment options. Further follow‐up and evaluation of a larger cohort are required to assess the response rate and durability of resumed responses and to identify determinants of response to rituximab and the relative safety and efficacy of other anti‐CD20 antibodies in this context.

## AUTHOR CONTRIBUTIONS

Study conception and design: Sasanka Handunnetti, Mary Ann Anderson, Andrew W. Roberts, Shuo Ma, Michelle Boyer, and John F. Seymour. Provision of study materials or patients: Sasanka Handunnetti, Mary Ann Anderson, Andrew W. Roberts, Matthew S. Davids, Shuo Ma, and John F. Seymour. Collection and assembly of data: Sasanka Handunnetti, Shuo Ma, Michelle Boyer, Jennifer Arzt, John F. Seymour, Abdullah Al Masud, Relja Popovic, Amanda Jacobson, and Su Y. Kim. Analysis and interpretation of the data: Sasanka Handunnetti, Mary Ann Anderson, Andrew W. Roberts, Matthew S. Davids, Shuo Ma, Michelle Boyer, Jennifer Arzt, Abdullah Al Masud, Relja Popovic, Amanda Jacobson, Su Y. Kim, and John F. Seymour. Writing, editing, and approval of the manuscript: Sasanka Handunnetti, Mary Ann Anderson, Andrew W. Roberts, Matthew S. Davids, Shuo Ma, Michelle Boyer, Jennifer Arzt, Abdullah Al Masud, Relja Popovic, Amanda Jacobson, Su Y. Kim, and John F. Seymour.

## FUNDING INFORMATION

Sasanka Handunnetti was paid honoraria from Gilead and Roche, got travel expenses from AbbVie, and non‐financial assistance from Novartis. Mary Ann Anderson is employed at Walter and Eliza Hall Institute of Medical Research, which receives milestone and royalty payments related to venetoclax (Dr. Mary Ann Anderson receives a financial benefit from these payments as a result of previous research related to venetoclax); got research funding from AbbVie and Genentech. Andrew W. Roberts is employed at Walter and Eliza Hall Institute of Medical Research, which receives milestone and royalty payments related to venetoclax (Dr Andrew W. Roberts receives a financial benefit from these payments as a result of previous research related to venetoclax); got research funding from AbbVie, Genentech, BeiGene, Janssen, and Servier; is an unremunerated advisor to AbbVie Australia. Matthew S. Davids is in the consultancy/advisory role for AbbVie, Adaptive Biotechnologies, Ascentage Pharma, AstraZeneca, BeiGene, Celgene, Genentech, Gilead Sciences, Janssen, MEI Pharma, Pharmacyclics, Syros Pharmaceuticals, TG Therapeutics, Verastem, and Zentalis; got research funding from Ascentage Pharma, AstraZeneca, Bristol Myers Squibb, Genentech, MEI Pharma, Pharmacyclics, Surface Oncology, TG Therapeutics, and Verastem. Shuo Ma is in the consultancy/advisory role/lecturing for AbbVie, AstraZeneca, BeiGene, Genentech, Gilead, Kite, Janssen, and Pharmacyclics; got institutional research funding from AbbVie, Acerta, BeiGene, Juno, NCCN, Novartis, Pharmacyclics, TG Therapeutics, and Janssen. Michelle Boyer is employed at and has the equity in F. Hoffmann‐La Roche Ltd. Jennifer Arzt, Abdullah Al Masud, Relja Popovic, and Su Y. Kim are employed at AbbVie and may hold stock or other options. Amanda Jacobson is currently employed at Pfizer and may hold stock or other options; was formerly employed at AbbVie and may hold stock or other options. John F. Seymour is in the consultancy/advisory role for AbbVie, AstraZeneca, Celgene, Gilead, Janssen, MEI Pharmaceuticals, Nurix, Roche, and Genentech; got research funding from AbbVie, Celgene, Janssen, and Genentech. Venetoclax is being developed in collaboration between AbbVie and Genentech Inc. AbbVie and Genentech funded the study and participated in the study design, research, analysis, data collection, and interpretation of data, as well as the writing, review, and approval of the publication. No honoraria or payments were made for authorship. Medical writing support was provided by Allison Cherry, PhD of Bio Connections, LLC, funded by AbbVie.

## Supporting information

Supporting information.

## Data Availability

This clinical trial data can be requested by any qualified researchers who engage in rigorous, independent scientific research, and will be provided following review and approval of a research proposal and Statistical Analysis Plan and execution of a Data Sharing Agreement. Data requests can be submitted at any time, and the data will be accessible for 12 months, with possible extensions considered. For more information on the process, or to submit a request, visit the following link: https://www.abbvie.com/our‐science/clinical‐trials/clinical‐trials‐data‐and‐information‐sharing/data‐and‐information‐sharing‐with‐qualified‐researchers.html.
