# Supplementary material for: Addition of rituximab in relapsed/refractory chronic lymphocytic leukemia after progression on venetoclax monotherapy
Source: EJHaem. 2021 Mar 3;2(2):266–71. doi: 10.1002/jha2.177 (PMC9175959; doi:10.1002/jha2.177)
Supplement: Supplementary file 1 — Supporting information. [file JHA2-2-266-s001.docx]

**Supplemental Data**

**Patient case reports**

*Patient A (*TP53*-mutated CLL)*

1. Patient achieved a minimal residual disease (MRD)-negative complete response (CR) on 800-mg venetoclax monotherapy (reduced to 600 mg after 3.3 months). Progressive disease (PD; increased disease in marrow) occurred after 67.1 months, with persistence of *TP53* mutation and emergence of new *SF3B1* and *BCL2* mutations.^1^ Patient received 6 cycles of rituximab with ongoing venetoclax, which restored MRD-negative CR. Patient maintains MRD-negative CR at 5.9-month follow-up.
2. *Patient B (11q-deleted CLL)*

Patient achieved an MRD-positive PR on 400-mg venetoclax monotherapy. After 56.2 months, peripheral blood (PB) MRD increased to a range of 0.26-0.98% over approximately 9 months. Following 6 cycles of rituximab and venetoclax dose escalation to 600 mg, partial response (PR) was achieved with undetectable MRD (uMRD) in PB, and patient maintains response at 5.6months of follow-up.

*Patient C (SLL)*

Patient achieved a PR on 400-mg venetoclax monotherapy; PD (lymphadenopathy) occurred after 38.2 months. Following completion of 6 cycles of rituximab, PR was achieved (MRD unavailable). At 5.4 months after rituximab, patient maintained ongoing PR; however, shortly after the data cutoff, the patient progressed and discontinued study.

*Patient D (fludarabine-refractory CLL)*

1. Patient achieved MRD-positive PR on 400-mg venetoclax monotherapy; PD (lymphadenopathy and increase in BM MRD) occurred after 44 months. Venetoclax was increased to 600 mg after 3 months of maintained SD. Patient received 6 cycles of concurrent rituximab 3 months later, and a PR (BM MRD+) was achieved. PD recurred 9 months after rituximab. Patient is currently being re-treated with an additional 6 cycles of rituximab and has PB uMRD after data cutoff.

*Patient E (11q-deleted,* TP53*-mutated CLL)*

1. Patient achieved an MRD-positive PR on 400-mg venetoclax but developed PD (lymphadenopathy) after 30.2 months, then received 6 cycles of concurrent rituximab. Patient had stable disease (SD) for 4.7 months on venetoclax after rituximab until trial cessation due to clinical disease progression.

*Patient F (fludarabine-refractory,* IgVH*-unmutated CLL)*

1. Patient achieved MRD-positive CR on 300-mg venetoclax and progressed after 67.7 months. Bone marrow (BM) demonstrated morphological recurrence of CLL but had concurrent therapy-related myelodysplasia contributing to cytopenias. After 5 cycles of concurrent rituximab, no objective response was achieved. The patient received no formal response assessment as of the data cutoff; however, The patient experienced ongoing clinical benefit, per investigator assessment, and remains on venetoclax monotherapy, with SD 15.1 months after rituximab.

*Patient G (fludarabine-refractory,* IgVH*-unmutated CLL)*

1. Patient achieved a PR on 400-mg venetoclax and progressed (lymphadenopathy) after 32.9 months. The patient received 6 cycles of concurrent rituximab; SD was best response without clinical benefit (7.5 months after rituximab). Patient discontinued study.

*Patient H (11q-deleted CLL)*

1. Patient achieved MRD-negative CR on 400-mg venetoclax. PD (lymphadenopathy) occurred 44.0 months later and, following 6 cycles of concurrent rituximab, no objective response was achieved. Patient discontinued study.

*Patient I (11q-deleted CLL)*

Patient achieved MRD-negative PR on venetoclax (200 mg) with rituximab. Venetoclax dose was increased to 400 mg after 29 months. PD (lymphadenopathy) occurred after 55.2 months, with increased lymphadenopathy on CT scans, and venetoclax was increased to 600 mg. Rituximab was restarted, and patient achieved PR 3 months later, followed by MRD-negative CR 12 months later. Patient remains on venetoclax 600 mg, with MRD-negative status.

*Patient J (11q-deleted CLL)*

Patient achieved MRD-positive PR on venetoclax (300 mg) with rituximab. Venetoclax dose was increased to 400 mg after 24 months. After PD with lymphadenopathy and PB lymphocytosis following 36 months on therapy, venetoclax was increased to 600 mg and patient restarted rituximab without response. Patient discontinued study due to PD.

**References**

1. Blombery P, Anderson MA, Gong JN, Thijssen R, Birkinshaw RW, Thompson ER, et al. Acquisition of the Recurrent Gly101Val Mutation in BCL2 Confers Resistance to Venetoclax in Patients with Progressive Chronic Lymphocytic Leukemia. Cancer Discov. 2019;9(3):342-53.
